# Intimate partner violence and challenges facing women living with HIV/AIDS in accessing antiretroviral treatment at Singida Regional Hospital, central Tanzania

**DOI:** 10.3402/gha.v9.32307

**Published:** 2016-12-15

**Authors:** Agnes Kosia, Deodatus Kakoko, Ave Maria Emilius Semakafu, Tumaini Nyamhanga, Gasto Frumence

**Affiliations:** 1 Department of Development Studies, School of Public Health and Social Sciences, Muhimbili University of Health and Allied Sciences, Dar-es Salaam, Tanzania; 2 Department of Behavioural Sciences, School of Public Health and Social Sciences, Muhimbili University of Health and Allied Sciences, Dar-es Salaam, Tanzania

**Keywords:** IPV, HIV/AIDS, challenges, women living with HIV/AIDS, antiretroviral drugs

## Abstract

**Background:**

Human immunodeficiency virus (HIV) remains a global public health problem. Sub-Saharan Africa is the region most affected by HIV/AIDS in the world. Globally, and in Tanzania in particular, women are more affected by HIV/AIDS than men. Tanzania has been reported to be among the countries with the highest burden of intimate partner violence (IPV). This study explored the challenges facing women living with HIV/AIDS (LWHA) attending the care and treatment clinic (CTC) in Singida Regional Hospital in Tanzania.

**Design:**

A qualitative study was performed in which data were collected through in-depth interviews with 35 women LWHA who also experienced IPV. Content analysis was used to analyse the data.

**Results:**

The study findings showed that women LWHA experienced challenges from their male partners in the form of lack of fare to attend CTC, delayed attendance to CTC, verbal threats and intimidation, mistrust partner resulting in changed antiretroviral (ARV) dosing time. Also, systemic challenges such as malfunction of CD4 count testing apparatus contributed to mistrust from their male partners which led to IPV.

**Conclusion:**

In this study, women LWHA experienced IPV challenges that resulted in poor adherence to ARV medication and CTC attendance, as well as insufficient time to collect ARV medication. It is recommended that the government address systemic challenges faced by women LWHA, introduce multiple approaches to address the needs of women LWHA experiencing IPV, and develop strong policies to prevent IPV against women in Tanzania, regardless of their HIV status.

## Introduction

Human immunodeficiency virus (HIV) remains a global public health problem (1). At the end of 2013, there were 35 million people living with HIV (PLHIV). This number is rising, because the use of antiretroviral (ARV) treatment means that more people with HIV are living longer (2). Sub-Saharan Africa (SSA) remains the region most affected by the HIV epidemic, according to the 2014 UNAIDS Gap report (2–4).

It is estimated that 86% of PLHIV in SSA know their disease status and are receiving ARV treatment (2, 5, 6). Since 1995, ARV treatment has prevented 7.6 million deaths globally, including 4.8 million deaths in SSA. These life-saving medicines have gained approximately 40.2 million life years and increased quality of life since the HIV epidemic started (1, 2). Women bear the burden of the HIV epidemic because of increased vulnerability and risk of HIV infection among adolescent girls and young women (2, 4).

### The cycle of intimate partner violence (IPV) and HIV infection

Studies show that there is an association between violence against women and the risk of HIV infection. Women in violent relationships have four times the risk of contracting sexually transmitted infections (STIs), including HIV, than women in relationships without violence. HIV serostatus disclosure may be an initiating or contributing factor for partner violence. HIV-positive women may experience abuse that is more frequent and more severe (6).

### IPV in Tanzania

In Tanzania, many types of violence are reported, and all have a negative impact on individuals and society, especially on women and children. IPV is perpetrated against women by their husbands or intimate partners (7, 8). Violence against HIV-positive women plays a crucial and devastating role in preventing access to ARV treatment (7). IPV against HIV-positive women has been reported to represent a significant obstacle to HIV prevention and treatment (8). Women living with HIV/AIDS (LWHA) are 10 times more likely to experience IPV than HIV-negative women. Furthermore, HIV-positive women are frequently blamed for transmitting HIV infection to their male partners (8). Maman et al. conducted a study in Dar-es Salaam and showed that violence and threats of violence are emerging factors in the increasing HIV epidemic among women. Violence increases a woman's risk of becoming HIV infected through forced or coercive sexual intercourse, thereby limiting her ability to negotiate HIV-preventive behaviours (9).

### HIV/AIDS situation in Tanzania

Tanzania has reported some success in controlling the HIV epidemic over the past decade. Scaling up access to ARV treatment has allowed the national impact of HIV to be minimised. Between 2010 and 2013, the country contributed 5% to the global total number of people newly accessing treatment. As a result, between 2005 and 2013, the number of people dying from AIDS-related illness decreased by 44%, and the total number of PLHIV in Tanzania declined from 7 to 5.1% from 2003/04 to 2011/12 (2, 10). According to the National AIDS Control Program Care and Treatment report, it is estimated that 21–30% of PLHIV in Tanzania have registered at care and treatment clinics (CTCs), and that 63–83% of those eligible for treatment are receiving ARV medication. The availability of ARV treatment has a significant impact on prolonging the lives of HIV-infected people (11, 12). Little is known about the challenges facing women LWHA as victims of IPV in Tanzania. This study examined the challenges facing women LWHA attending CTC clinics in the Singida Regional Hospital in Tanzania.

## Methods

### Study area

We conducted a study at the CTC and prevention of mother to child transmission (PMTCT) of the HIV/AIDS unit under the reproductive and child health (RCH) unit at Singida Regional Hospital. According to the 2012 Tanzania HIV/AIDS and Malaria Indicator Survey (THMIS), the prevalence of HIV in Singida is 3.3%. Similarly, according to Tanzania DHS 2010, the Singida region has been reported to have a high prevalence of IPV. Physical violence was 50.7%, sexual violence was 21.8% and emotional violence was 50.7% (13). The Singida region is among the eight regions in Tanzania that have reported an increase in the prevalence of HIV/AIDS; women are more affected than men, with 6.6% prevalence versus 2.8%, respectively (14).

### Study design

This was a qualitative study in which data were collected through in-depth interviews (IDIs). The use of IDIs enabled the researcher to gain an in-depth experience of victims of IPV who are HIV-positive. Due to the nature of the study, the researcher opted for the ‘snowballing’ method in order to easily identify eligible participants who were difficult to locate.

The inclusion criteria/eligibility for participation was defined as being a woman LWHA who had ever had a male partner and experienced IPV. Widows were included in order to get their retrospective experience on the impact of IPV on ARV use. The risk/implication of using snowballing techniques is that they work by chain referral, meaning that people are more likely to identify those with similar issues of interest.

### Selection of study participants

Purposive sampling was used to get a sample of women LWHA who experienced IPV.

The CTC and PMTCT clinics were used as an entry point where women LWHA were identified. The counsellors introduced the researcher to the first few respondents, who were known to have experienced IPV. Through the snowballing technique, the initial respondents were requested to identify others who had similar experiences.

The snowballing method was initiated by interviewing one woman. The interviewed woman was then asked to identify other women who were HIV-positive and experiencing IPV from their male partners. Widows were asked to give their retrospective accounts of what happened when their partners were alive. Among widowed and separated women, some were living with new partners while others had decided to live alone with their children. The majority of HIV-positive women knew each other through the use of CTC services and attendance at support groups, which met once a month to provide psychosocial support.

### Data collection

IDIs were used to collect data; this involved conducting intensive individual interviews to explore the challenges facing victims of IPV. The interviews were conducted by the first author (AK) between February and March of 2014. The supervisors took part in the analysis process, in which they constructively reviewed categories and themes that merged from the data. A total of 35 interviews were conducted. Interviews were conducted in the Kiswahili language and transcripts were later translated into English for the purpose of quoting the participants. Each interview lasted between 45 and 60 min. Prior to the interviews, the interviewer introduced herself to each participant, explained the purpose and importance of the study, and assured her that all of the information provided would be handled carefully and that confidentiality would be maintained throughout. Permission for note-taking and audio recording was requested and granted by the participants.

### Data analysis

The audio-recorded interviews were transcribed by the first author and translated from Kiswahili into English. A sample of translated interviews was back-translated to Kiswahili to ensure that the translation had been done correctly and accurately. The transcriptions were verbatim in Kiswahili. So the verbatim transcriptions were only translated into English to help the researcher during writing. The meaning of the participants’ experience was maintained because the translation was done by two people, and comparison was made to ensure that the original meaning was maintained. The transcripts and field notes were analysed manually by reading and re-reading the text to ensure familiarity with the data. Similarities and differences were examined to determine the relationship between the codes and the meaning. Content analysis was used in a wide range of analytical approaches to analyse the data, and jargon was avoided. Back-and-forth review of the text ensured that appropriate units, codes, and themes were generated to interpret the latent content and thereby respond to specific research questions.

### Ethical considerations

Ethical clearance was granted by the Muhimbili University of Health and Allied Sciences (MUHAS). Permission to conduct the study in the region was obtained from the Singida Regional Authorities, including the regional administrative secretary, regional medical officer, and the Singida Regional Hospital managers. All interviewees provided written informed consent to participate in the study.

## Results

### Socio-demographic characteristics of the study participants

The participants in this study were women LWHA attending CTC in the Singida region, and from the Nyaturu and Nyiramba tribes only. Participants were aged between 20 and 79, and the majority were widows as their husbands had died from HIV/AIDS. Among the remainder, most were married, although a few had separated from their male partners due to violence. Few women interviewed were cohabiting. The majority of participants had undergone formal education, although few had primary or secondary school education. Economically, the majority of study participants were engaged in small businesses such as selling local brew or fruits and tailoring, whereas others were peasants (Table 1).

**Table 1:** Basic characteristics of study participants

Category	HIV-positive women	Number (*n*=35)
Age	20–39	16
	40–59	18
	60–79	1
Marital status	Married	10
	Widowed	16
	Separated	7
	Cohabiting	2
Level of education	No education	13
	Primary school	16
	Secondary school	6
Source of income	Small business	20
	Peasant	15

The analysis of the IPV challenges generated six themes: lack of fare to attend CTC, delayed attendance at CTC, verbal threats and intimidation from male partner, mistrust by a male partner, malfunction of CD4 count testing apparatus, and changed ARV dosing time.

### Lack of fare to attend CTC

Participants in this study reported that lack of bus fare due to poor economic status made them miss their CTC clinic appointments. Married participants reported that their male partners did not provide them with the money required to take a bus to attend CTCs, so that they ended up missing their CTC appointments. According to participants, male partners were able to afford the fare, which was only one dollar (2,000 Tanzanian shillings) for a return trip. In these situations, some participants received financial assistance from their relatives but had to attend the CTC on a different day to their appointment date. As a result, they had to queue for a long time to wait for those who had appointments to be seen first, as those who came without an appointment were seen last. This was narrated by one participant, who said:

I went to the CTC on a day which was not my clinic because I missed my appointment day due to lacking the fare for the bus. (Married woman, aged 32)

Furthermore, in our study, widowed women LWHA reported that, during their relationship with their late husbands, they lacked the money needed to buy food to sustain their health and had to engage in small business to ensure financial assistance. When they requested money from their husbands to buy food, they received insults from their male partners, as described by one participant:

There was a time I was very sick and I laid down for sometimes because of my sickness. I didn't have any money to buy food for my children and myself and even the fare to attend my clinic. I asked my late husband if he could help me with that, but my husband never gave me money to attend the HIV clinic. (Widowed woman, aged 45)

### Delayed attendance at CTC

In our study, married women LWHA reported that they had to request permission in advance to attend CTC appointments. The majority reported that, on the day of the appointment, their male partners were violent as they associated the appointment with a lack of care/abandonment by their female partners. Therefore, to ensure that they did not suffer on the day of the CTC appointment, male partners provided a long list of domestic activities for women to perform prior to attending their appointment. Such activities included washing their husbands’ clothes, fetching enough water for domestic use, picking up children from school, and preparing meals on time. They therefore missed morning CTC sessions, including information on adherence to ARV medications and clinic appointments, the side effects of ARV medication, and the importance of nutrition for women LWHA. This was described by one participant:

On the day of my clinic, it happens that I was late attending the CTC because my husband gave me a long list of activities that I had to do before I attended. If I don't do these, he will not allow me to attend the clinic and I don't want to miss it. I arrived at the CTC late and missed all of the morning sessions. (Woman, aged 44)

### Verbal threats and intimidation from male partner

In our study, married women LWHA reported that they experienced verbal abuse from their male partners. They were insulted with rude words; they were told that they are dead, walking useless people who would die soon. They were shouted at when they were seen their health become worse. Male partners were not happy to see participants with tins of ARV medications in their bedrooms, and some got angry and threw the medications into pit latrine toilets so that they could no longer be accessed. Therefore, participants ran short of ARV medications and poor adherence to treatment. This was described by one of the participants:

My man doesn't want to see me taking my ARV medication in our bedroom. He took my pills and threw them into the toilet so that I can't access them anymore. I ran short of my medication and I was scared to go back to the hospital to get another dose. (Married woman, aged 32)

### Mistrust by a male partner

Findings revealed that widowed women LWHA experienced IPV during their relationship with their late husband or partners as they faced suspicion from their male partners. The mistrust arose as a result of the systemic challenges that they faced at the CTC, such as an absence of ARV stock out, long waiting times, and limited functionality of CD4 count testing apparatus. With regard to ARV medication, it was reported that CTCs had frequent shortages of these drugs, resulted into supply of 2-weeks instead of a 4-week supply. As a result of this shortage, patients were required to return for the remaining medication after 2 weeks which caused mistrust by male partner among women LWHA. This was described by one participant:

I told my late husband that I was late arriving home because the clinic was full of patients, so we had to wait a long time to see the Dr, collect our medication, and receive our CD4 results. He didn't believe me and thought I was having an affair with another man. (Widowed woman, aged 42)

### Malfunction of CD4 count testing apparatus

Malfunctioning apparatus for testing CD4 counts at the CTC meant that women LWHA were unable to have routine checks of their CD4 counts. CD4 apparatus functionality was typically sporadic, and machines took 1–2 months to get fixed. As a result, women LWHA had to request permission and money from their male partners to test their CD4 counts at the nearest hospital, which was 25 km away from the CTC. These situations resulted in IPV and separation for women LWHA, as expressed by one participant:

At the CTC, the CD4 machine was not working most of the time. Every single visit I went I was always told that it was not working or was under maintenance. To me, it was very cumbersome because when I think about requesting permission to test my CD4 at another hospital, which far away and costs money, it caused misunderstandings between me and my husband. (Separated woman, aged 28)

## Changed ARV dosing time

Women reported that, regardless of the challenges they were facing from their male partners in relation to adherence to ARV treatment, they did not stop taking their medication as they understood the importance of good adherence to CTC attendance and ARV treatment. They reported hiding their ARV medications in places where their male partners could not access them. Others reported changing their ARV medication dosing times to a time when they knew that their male partners would not be able to see them taking their medication. In this way, they managed to adhere to ARV treatment. These actions allowed participants to improve their health, thereby increasing their productivity.

I have never missed my dose. I found a different time when I can take my medication, without missing my doses. Now I never miss a single dose of drugs because those medicines help me a lot. They keep me healthy and I can do my daily activities, which helps me to maintain my family life, such as paying rent and school fees for my children and buying food for my kids. (Separated woman, aged 32)

## Discussion

The study examined IPV and challenges facing women LWHA in accessing ARVs at Singida Regional Hospital in central Tanzania, as a perception of women LWHA experiencing IPV in Tanzania is not yet well documented. We hope that the information in our study will contribute to improving systemic and social challenges facing women LWHA experiencing IPV in accessing their ARVs at CTCs. This section is organised into six subsections. The first section discusses the lack of fare to attend CTC, the second delayed attendance at CTC, the third verbal threats from male partners, the fourth mistrust by a male partner, the fifth changed ARV dosing time, and the sixth malfunction of CD4 testing apparatus.

In our study, we found that women LWHA could not attend CTC and PMTCT appointments on time because of a lack of bus fare. This financial limitation could be attributed to low socio-economic status, less profitable small-scale businesses such as tailoring, selling local brews, and domestic activities. Some reported that their husbands denied them financial support. Our findings are in line with a study done in Malaysia, which reported that social barriers to adherence, such as difficulty in paying for transport, were among the challenges to ART treatment adherence among women LWHA (10). This implies that a lack of financial independence, which is compounded by the experience of IPV, hinders women's access to ARV treatment. Similar studies done in Dar-es Salaam (11) and Botswana (12) found that women LWHA fail to attend CTC due to a lack of fare for transport. Thus, they run short of ARVs, which is associated with poor adherence to ARV treatment, which results in treatment failure due to a lack of fare to attend CTC.

Those who obtained financial support from their relatives to attend their appointment had to queue at CTC for a long time and were served last because they attended the CTC on different days which resulted in long waiting times/led to IPV from their male partners. Also, a long distance and unaffordable travel cost were associated with challenges in accessing ARVs among women LWHA in Uganda (12).

In our study, the majority of women reported that, on the day of the appointment, their male partners were violent, as they associated the appointment day with a lack of care/abandonment by their female partners. Our study is similar to a study done in eastern Uganda on IPV against women and implications for HIV prevention, which reported that men complained that their female partners who attend CTC neglected house work, went out without permission and returned home late.

Our study is in line with studies done in Mozambique (15) and Zambia, which showed that men's fear and denial of HIV have interfered with women's adherence to ARV treatment (16). Furthermore, the study done in Tanzania showed that women LWHA are at high risk of violence because of the behaviours of their male partners, which are associated with cultural and norms issues (9).

Mistrust from their male partner was interpreted as the belief that women attending CTC take the opportunity to have extramarital relationships, which resulted in mistrust from their male partners. We also found that women LWHA's lack of trust was caused by challenges at the treatment clinic, such as lack of ARV medication stocks, too many patients at the CTC, and shortage of health care workers, leading to slow service and excessive waiting times and, subsequently, exacerbated IPV among women LWHA. Our findings correlate with those from Uganda, which showed that a long waiting time at the CTC was associated with wastage of earnings (17).

In our study, women LWHA reported that their male partners were not happy seeing them taking ARVs in front of them, as it was associated with travel to the CTC for ARV refill and adherence. To avoid conflict with their male partners, the women had to change their ARV dosing time. Our finding are in line with a study done in Zambia, which found that a lack of decision-making among HIV-positive women experiencing IPV has been attributed to a lack of constant refill of ARVs, which is associated with travel and other potential expenses, which have been reported to be barriers among HIV-positive women who lack knowledge, permission, and support from their male partners (18).

Malfunctioning of CD4 testing apparatus was also reported as a challenge in our study. Women LWHA reported that CD4 machines were not working most of the time and could take a long time to be fixed; as a result, they were unable to test their CD4 counts, a measure which is very important for patient monitoring. This observation is in accordance with a systematic review in Malaysia, which showed that a lack of CD4 testing and delays in receiving CD4 results were contributing factors to HIV-positive patients becoming lost to care in SSA (19, 20).

### Strengths and limitations of the study

Trustworthiness was enhanced by ensuring credibility, transferability, dependability, and confirmability of the study (21). Credibility was ensured in four main ways: firstly, the researchers requested respondents to be honest by telling them that there were neither right nor wrong answers and that all answers were valuable; secondly, the researchers used probes to elicit detailed information and iterative questioning, which involved rephrasing questions in order to clarify issues; thirdly, member check technique was used, which involved the researchers asking the respondents if the recorded words matched what they actually intended; and, finally, by conducting frequent debriefing sessions involving multidisciplinary research team to reflect and discuss procedures in the course of data collection and interpretation of the results, we strove to ensure confirmability and consistency.

Another aspect of trustworthiness in this study is transferability – which refers to application of the findings in a similar situation. This was ensured through a clear description of the knowledge gap and on how the study was carried out. The detailed description of the study methods also served to ensure dependability. In particular, the purposive sampling of study participants aimed at facilitating transferability of the study findings.

Nevertheless, this study suffered two limitations. 1) The fact the study relied on IDIs as the main data collection limited triangulation of the study findings. This was mitigated by complimenting interview transcripts with field notes. 2) Snowball sampling might have resulted into a bias of recruiting participants with similar IPV experiences which might have led to overstating negative experiences, thereby compromising objectivity of the findings. The above-described measures which were taken to ensure credibility also served to minimise the bias (22, 23).

## Conclusions

Women LWHA experienced IPV chaos from their male partners which was characterised by a state of total confusion with no order, disruption, unpredictable behaviour, and unorganized life. Lack of male support led to poor adherence to ARV medication, and CTC systemic challenges, such as the absence of ARV medication stocks and inadequate functionality of CD4 testing apparatus, led to exacerbated IPV. We strongly recommend that the government of Tanzania address the systemic challenges faced by women LWHA experiencing IPV so that they can access the appropriate CTC services to prevent IPV. The government should introduce policies to provide multiple approaches to address the needs of women LWHA experiencing IPV such as a supportive environment, involving women LWHA as peer educators to reach more women with support and a network. IPV against women LWHA should be incorporated into various programmes to address the overlapping of the HIV epidemic and violence against all women in Tanzania, regardless of their HIV status.

## Paper context

Intimate partner violence (IPV) against HIV-positive women has been reported to be a significant obstacle to HIV prevention and treatment. This paper focuses on challenges facing women living with HIV/AIDS (LWHA) as victims of IPV. The paper provides lived experiences of interplay between IPV and utilisation of ARVs among HIV-positive women in Singida. We recommend to the government of Tanzania to address systemic challenges faced by women LWHA in accessing HIV care and treatment.
